# Methane potentials of wastewater generated from hydrothermal liquefaction of rice straw: focusing on the wastewater characteristics and microbial community compositions

**DOI:** 10.1186/s13068-017-0830-0

**Published:** 2017-05-31

**Authors:** Huihui Chen, Cheng Zhang, Yue Rao, Yuhang Jing, Gang Luo, Shicheng Zhang

**Affiliations:** Shanghai Key Laboratory of Atmospheric Particle Pollution and Prevention (LAP3), Department of Environmental Science and Engineering, Fudan University, Shanghai, 200433 China

**Keywords:** Hydrothermal liquefaction wastewater, AD, Microbial community compositions, Methane yields, Organic compositions

## Abstract

**Background:**

Hydrothermal liquefaction (HTL) has been well studied for the bio-oil production from biomass. However, a large amount of wastewater with high organic content is also produced during the HTL process. Therefore, the present study investigated the methane potentials of hydrothermal liquefaction wastewater (HTLWW) obtained from HTL of rice straw at different temperatures (170–320 °C) and residence times (0.5–4 h). The characteristics (e.g., total organic content, organic species, molecular size distribution, etc.) of the HTLWW were studied, and at the same time, microbial community compositions involved in AD of HTLWW were analyzed.

**Results:**

The highest methane yield of 314 mL CH_4_/g COD was obtained from the sample 200 °C–0.5 h (HTL temperature at 200 °C for 0.5 h), while the lowest methane yield 217 mL CH_4_/g COD was obtained from the sample 320 °C–0.5 h. These results were consistent with the higher amounts of hard biodegradable organics (furans, phenols, etc.) and lower amounts of easily biodegradable organics (sugars and volatile fatty acids) present in sample 320 °C–0.5 h compared to sample 200 °C–0.5 h. Size distribution analysis showed that sample 320 °C–0.5 h contained more organics with molecular size less than 1 kDa (79.5%) compared to sample 200 °C–0.5 h (66.2%). Further studies showed that hard biodegradable organics were present in the organics with molecular size higher than 1 kDa for sample 200 °C–0.5 h. In contrast, those organics were present in both the organics with molecular size higher and less than 1 kDa for sample 320 °C–0.5 h. Microbial community analysis showed that different microbial community compositions were established during the AD with different HTLWW samples due to the different organic compositions. For instance, *Petrimonas*, which could degrade sugars, had higher abundance in the AD of sample 200 °C–0.5 h (20%) compared to sample 320 °C–0.5 h (7%). The higher abundance of *Petrimonas* was consistent with the higher content of sugars in sample 200 °C–0.5 h. The higher *Petrimonas* abundance was consistent with the higher content of sugars in sample 200 °C–0.5 h. The genus *Syntrophorhabdu*s could degrade phenols and its enrichment in the AD of sample 320 °C–0.5 h might be related with the highest content of phenols in the HTLWW.

**Conclusions:**

HTL parameters like temperature and residence time affected the biodegradability of HTLWW obtained from HTL of rice straw. More hard biodegradable organics were produced with the increase of HTL temperature. The microbial community compositions during the AD were also affected by the different organic compositions in HTLWW samples.

**Electronic supplementary material:**

The online version of this article (doi:10.1186/s13068-017-0830-0) contains supplementary material, which is available to authorized users.

## Background

Due to the challenge of energy security, demand of bioenergy (derived from biomass) has been growing very fast in recent decades. Biomass can be converted into fuels (e.g., bio-oil, methanol, ethanol, biodiesel, etc.) and valuable chemicals (e.g., xylose, phenols, etc.) by various physicochemical and biological methods [1, 2]. Hydrothermal liquefaction (HTL) is an option to generate renewable bio-oil from biomass. Since water works as solvent in the HTL process, a large amount of wastewater with high concentrations of both organics and nutrients are produced [3, 4]. Previous studies mainly focused on the characterization and potential utilization of the bio-oil [5, 6], and little attention has been paid on the utilization of hydrothermal liquefaction wastewater (HTLWW) even though a significant fraction (20–50%) of the organic components in the biomass could enter HTLWW [7, 8].

Inappropriate disposal of HTLWW would result in environmental pollution considering its high organic contents. AD is a proven technique and widely used in the treatment of organic wastes/wastewater [9]. A previous study investigated the methane potential of HTLWW obtained from HTL of algae [4], and it was reported that around 44–61% of the COD was removed and converted to biogas by AD. However, the HTLWW in the above study was not well characterized. Our recent publication showed that the methane yield of HTLWW obtained from HTL of straw at 27 days can be enhanced by organic solvent extraction, and the HTLWW conversion efficiency was 53% [10]. A biohythane production [11] for high energy recovery and carbon recovery is 79.0 and 67.7%, respectively, from HTL cornstalk, but it was limited by low hydrogen fermentation performance. The long lag phase and even complete inhibition of methane production during AD of HTLWW obtained from HTL of swine manure was also reported [12]. Reasons for low conversion efficiency were the presence of recalcitrant organics formed during the HTL, while they were not well characterized.

Lignocellulosic biomass, which is different from algae in both chemical composition and structure, is abundant in the world [13]. Their HTL under various conditions (temperatures and resident times) for either bio-oil or carbon production has been studied previously [14, 15]. Lignocellulosic biomass is mainly composed of cellulose, hemicellulose, and lignin [16]. It has been reported that cellulose and hemicellulose could be fully hydrolyzed within the range of 150–270 °C [17, 18] forming sugar compounds and furan derivatives including HMF and furfural. Lignin had a two-phase mechanism reaction with a very broad temperature spectrum (>200 °C) [19]: very fast reaction of lignin fragments by breaking lignin-sugar polymers (mainly hemicelluloses) bonds into soluble fragments and slower reaction where the fragments react with one another (sugar and/or sugar degradation products such as furfural [18]) to produce phenolic compounds [20]. However, the organics in the HTLWW were not well characterized. Especially, the changes of organic compositions in the HTLWW with the changes of HTL conditions remains to be elucidated, which closely related with the anaerobic biodegradability of HTLWW. Understanding the organics in the HTLWW would facilitate the selection of HTL conditions to avoid the formation of recalcitrant organics. Different technologies have been applied to characterize the organics in wastewater originated from different sources (Landfill leachate, sludge hydrolysate, straw hydrolysate, etc.) [10, 21, 22]. For example, GC–MS was used to identify the main classes of molecules compositions, GC-FID was used to quantify the C_2_–C_6_ fatty acids, 3D-EEM fluorescence spectroscopy was used to identify fluorescent organics, and size distribution analysis was used to understand the physical characteristic of organics. The combination of the above-mentioned technologies can provide detailed information of organics in wastewater from different aspects, which has not been applied for HTLWW. In addition, the microorganisms which could degrade the organic compositions in HTLWW would be enriched in the AD. Therefore, the characterization of microorganisms involved in the AD of HTLWW obtained from various HTL conditions would improve our understanding of AD from microbiological aspect.

Rice straw is a major source of lignocellulosic biomass. Annually, around 731 million tons of rice straw is produced by Asia alone [23]. Therefore, the present study investigated the methane potentials of HTLWW from rice straw obtained at different temperatures (170, 200, 230, 260, 290, and 320 °C). Besides, sample 200 °C had the highest sugar content (hemicellulose and cellulose hydrolysate), which was easy to be biodegraded. Therefore, we studied different residence times at 200 °C (0.5, 1, 2 and 4 h). The combination of UV, 3D-EEM, GC-FID, GC–MS, and size distribution analysis were used to characterize the HTLWW in order to reveal the correlation between organics and methane potentials. Microbial communities established in the AD with different HTLWW samples were also investigated by high-throughput sequencing of 16S rRNA genes.

## Results

### Characteristics of HTLWW samples

COD and TOC were determined as they were two parameters that reflect the total organic content in wastewater. As shown in Table 1 and Additional file 1: Figure S1, the COD and TOC concentrations of HTLWW varied from 11.35 to 29.02 and 3.92 to 10.27 g/L, respectively, when the HTL temperatures varied from 170 to 320 °C. There were two peaks for COD and TOC values at 200 and 290 °C in the tested temperature range when the residence time was constant (0.5 h). The pH of all the HTLWW samples varied from 5.56 to 3.68, and it was due to the formation of organic acids, which was shown in the following part.

**Table 1:** pH, COD, TOC, and UV_245_ values of HTLWW samples under different HTL conditions

Parameters	170 °C–0.5 h	200 °C–0.5 h	230 °C–0.5 h	260 °C–0.5 h	290 °C–0.5 h	320 °C–0.5 h	200 °C–1 h	200 °C–2 h	200 °C–4 h
pH	5.56 ± 0.01	4.09 ± 0.02	3.81 ± 0.21	3.72 ± 0.07	3.69 ± 0.12	3.68 ± 0.07	3.86 ± 0.10	3.80 ± 0.02	3.81 ± 0.09
COD g/L	11.35 ± 1.19	27.58 ± 1.82	22.25 ± 2.05	24.33 ± 1.98	29.02 ± 1.09	24.33 ± 1.19	23.2 ± 1.04	14.28 ± 2.88	19.00 ± 3.29
TOC g/L	3.92 ± 0.03	9.99 ± 1.12	8.55 ± 0.12	8.88 ± 0.50	10.27 ± 1.04	9.23 ± 0.87	9.03 ± 0.32	5.18 ± 0.93	7.29 ± 0.02
Organic in HTLWW %^a^	12.11 ± 1.27	29.42 ± 3.08	23.74 ± 3.25	25.96 ± 2.11	30.96 ± 4.16	25.96 ± 1.26	24.75 ± 1.10	15.23 ± 3.07	20.27 ± 3.51
UV_254_/COD^b^	2.46 ± 1.19	2.57 ± 2.89	3.91 ± 3.05	2.48 ± 1.98	2.24 ± 3.90	1.99 ± 1.19	4.39 ± 1.04	4.63 ± 2.99	3.95 ± 3.29

^a^The percentage organics (in COD values) in HTLWW, COD of rice straw input in HTL process was calculated according to C, H, O, and N elemental compositions

^b^UV_254_ unit was 1/m

### Methane potentials of HTLWW samples

**Fig. 1:**
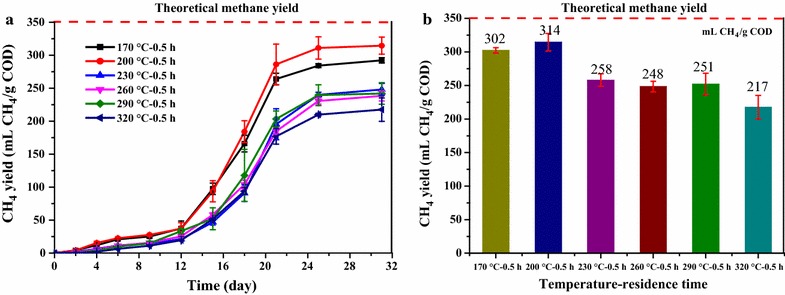
Methane yields of HTLWW obtained under different HTL temperatures: **a** time courses of methane production and **b** methane yields. The methane production from inoculum was subtracted for the calculation of the methane yields of different HTLWW samples

**Fig. 2:**
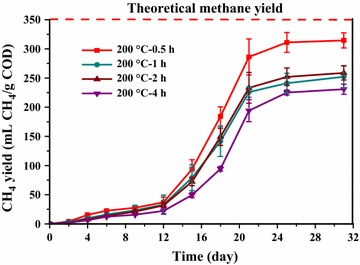
Methane yields of HTLWW obtained under different HTL residence times. The methane production from inoculum was subtracted for the calculation of the methane yields of different HTLWW samples

Figure 1a shows the time courses of cumulative methane production from HTLWW obtained from different temperatures with the residence time 0.5 h. The rapid increase of methane production was observed after 12 days of acclimatization, and maximum methane production was achieved after around 21 days of digestion. The methane yields (Fig. 1b) at the end of the experiments were 302, 314, 258, 248, 251, and 217 mL CH_4_/g COD (calculated at STP conditions) for samples 170 °C–0.5 h, 200 °C–0.5 h, 230 °C–0.5 h, 260 °C–0.5 h, 290 °C–0.5 h, and 320 °C–0.5 h, respectively. ANOVA analysis showed that there was significant difference in methane yields for all the HTLWW samples (*p* < 0.05). The time courses of cumulative methane production from HTLWW obtained from HTL at 200 °C with varying residence times are shown in Fig. 2. The increase of residence time from 0.5 to 1, 2 and 4 h resulted in the decrease of methane yield from 315 mL CH_4_/g COD to around 250 mL CH_4_/g COD.

### UV spectra analysis of HTLWW samples

**Fig. 3:**
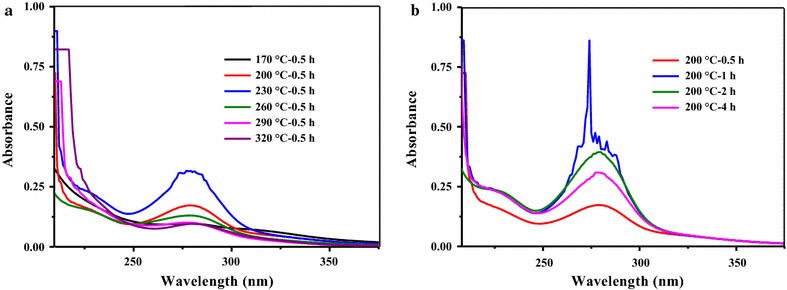
UV–Vis spectra of the HTLWW: **a** spectra at different liquefaction temperatures; **b** spectra at different residence times when temperature was 200 °C

Figure 3 shows the UV spectra of HTLWW samples obtained at various HTL conditions. All samples contained a single prominent peak at approximately 280 nm, shouldered at 320 and 250 nm. The peak values of absorbance at the range of 320–250 nm increased first when HTL temperature rose from 170 to 230 °C and then it decreased when the temperature was further increased to 320 °C. The absorbance of samples 170 °C–0.5 h, 290 °C–0.5 h, and 320 °C–0.5 h were similar, while sample 230 °C–0.5 h had the biggest absorbance. As Fig. 3b shows, the peak values of absorbance at the range of 320–250 nm was also affected by residence time, and the highest value was found for sample 200 °C–1 h at 280 nm.

### 3D-EEM analysis of HTLWW samples

**Fig. 4:**
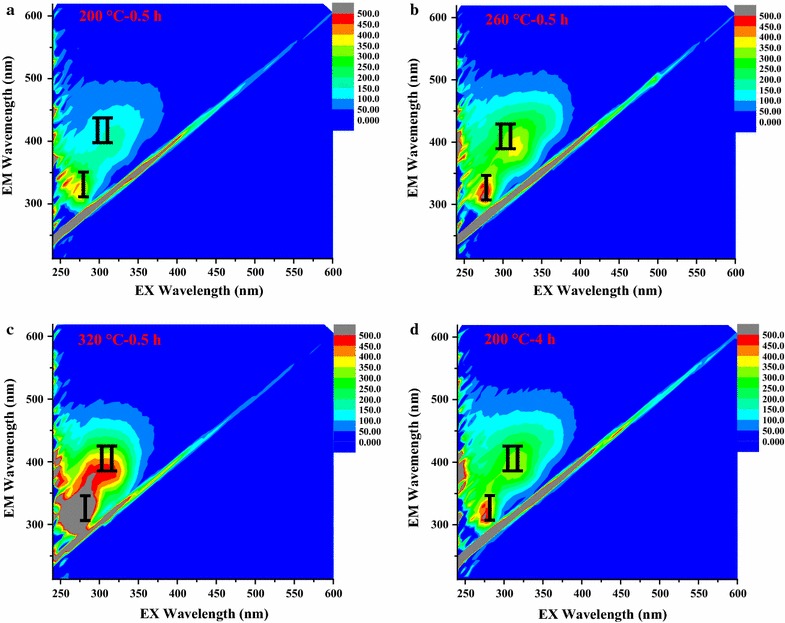
3D-EEM fluorescence spectrums of different HTLWW samples and (*I*) represents easily biodegradable compounds; (*II*) hard biodegradable organics such as phenols. **a** Sample 200 °C–0.5 h; **b** sample 260 °C–0.5 h; **c** sample 320 °C–0.5 h; **d** sample 200 °C–4 h

Samples 200 °C–0.5 h, 260 °C–0.5 h, 320 °C–0.5 h, and 200 °C–4 h were chosen for the analysis. Figure 4 shows fluorescent components and their relative concentrations of samples at 200, 260, and 320 °C. Fluorescent components were detected in all samples and increased when the temperature of HTL increased from 200 to 320 °C. Region of Ex/Em = 250–275/300–350 referred to accessible and easily biodegradable compounds such as fatty acids [24]. Fatty acids such as acetic acid (Ex/Em wavelength is 260/305) were verified to be produced even at lower temperature [25]. However, region Ex/Em = 280–325/380–425 nm was correlated to the hard biodegradable organics such as phenols like compounds [24, 25], which might be a notable reason for the low methane yield of sample 320 °C–4 h. The EEM spectra of samples 260 °C–0.5 h (Fig. 4b) and 200 °C–4 h (Fig. 4d) were similar which might indicate that the increase of either residence time or temperature of HTL could produce similar fluorescent compounds.

### GC–MS analysis of HTLWW samples

Table 2 shows the organic species in HTLWW samples identified by GC–MS. The organics were classified into acetic acid, alcohols, furfurals, ketones, and phenols. There were almost no volatile organic substances detected by GC–MS except little amounts of furans and acetic acid when HTL temperature was 170 °C. Rice straw was converted to alcohols (pentanol, butanol, and hexanol) and furans (furfural, methylfurfural, and 5-hydroxymethylfurfural) firstly when the temperatures were in the range of 170–230 °C, and more ketones (butanone, cyclopentenone, and hexanedione) were produced when the HTL temperature was increased from 170 to 230 °C.

**Table 2:** Relative abundances of organic species identified by GC–MS in the HTLWW samples based on peak areas

Name	170 °C–0.5 h	200 °C–0.5 h	230 °C–0.5 h	260 °C–0.5 h	290 °C–0.5 h	320 °C–0.5 h	200 °C–1 h	200 °C–2 h	200 °C–4 h
Acetic acid	5.90	4.21	4.18	2.93	3.62	3.82	2.42	4.98	4.12
3-Pentanol	–^a^	0.71	0.80	0.93	0.70	0.50	0.49	1.08	0.96
3-Methoxy-2-butanol	–	0.22	0.79	–	–	–	–	–	–
6-Methoxy-2-hexanol	–	0.35	0.74	–	–	–	–	–	–
Alcohols	–	1.28	2.32	0.93	0.70	0.50	0.49	1.08	0.96
2-Methylfuran	12.78	40.78	30.64	5.40	5.33	4.91	0.98	1.24	2.39
5-Methylfurfural	10.35	2.15	1.87	3.18	1.10	0.21	3.36	3.06	2.82
Furfural	23.13	42.93	32.51	10.46	7.19	2.57	57.04	47.86	33.33
2-Acetyl-5-methylfuran	–	–	–	1.53	1.10	1.27	1.01	0.94	1.05
5-Hydroxymethyl-2-furfural	–	–	5.31	4.40	–	–	7.73	5.68	6.60
Furans	46.25	85.87	70.33	24.98	14.71	8.95	70.12	58.79	46.19
Propanolone	–	2.56	5.89	4.63	0.99	0.54	5.26	4.49	5.47
Butanone	–	–	–	0.87	0.20	0.10	0.57	0.47	0.37
3-Hydroxy-2-butanone	–	2.18	2.08	1.74	1.71	1.40	4.92	3.65	5.92
2-Methyl cyclopentenone	–	–	–	7.39	12.19	6.45	1.83	1.66	2.63
3-Methyl cyclopentenone	–	–	–	2.07	3.65	7.64	–	–	0.99
2,3-Dimethyl cyclopentenone	–	–	–	1.54	4.51	4.27	–	0.49	0.53
2-Hydroxy-3-methyl cyclopentenone	–	–	2.32	4.05	4.00	1.05	1.68	1.88	3.35
2-Hydroxy-3-ethyl cyclopentenone	–	–	0.70	3.31	2.76	2.10	0.79	1.50	2.26
3-Ethyl cyclopentenone	–	–	–	1.35	1.58	3.15	–	0.62	0.75
2,3-Pentanedione	–	–	–	1.44	1.04	–	0.51	0.62	0.30
2,5-Hexanedione	–	–	2.79	5.55	4.94	2.97	2.13	2.04	3.36
3-Ethyl cycloheptanone	–	–	–	1.80	0.90	2.21	–	–	0.73
3-Octanone	–	–	–	1.33	0.98	1.03	0.88	0.77	1.07
1-Cyclopentyl-ethanone	–	–	–	1.29	1.13	1.00	0.53	0.67	0.88
1-(4-Hydroxy–3-methoxy-) acetophenone	–	–	–	0.92	0.57	0.75	–	1.01	0.84
1-(4-Hydroxy–3,5-dimethoxy-) acetophenone	–	–	–	2.68	2.70	2.13	1.16	1.91	1.55
1-(4-Hydroxy–3-methoxy-) propiophenone	–	–	–	1.27	0.83	1.16	–	0.82	1.29
4-Isopropyl- cyclohexenone	–	–	–	2.08	1.82	1.31	–	0.24	0.65
6-Methoxy-3-isopropyl-cyclohexanone	–	–	–	1.19	1.03	2.28	–	–	0.25
Ketones	–	4.74	13.79	46.48	47.54	41.57	15.76	29.35	33.18
Phenol	0.00	0.00	1.02	3.71	4.53	9.49	0.87	1.35	2.17
2-Methoxy-phenol	20.58	1.68	3.73	8.37	12.26	13.12	2.26	3.98	5.75
4-Ethyl-phenol	0.00	0.00	0.89	2.66	2.71	5.90	0.50	0.90	1.60
4-Ethyl-3-methoxy-phenol	15.84	1.29	1.59	3.08	3.16	3.20	1.19	2.77	2.71
2,6-Dimethoxy-phenol	11.44	0.93	2.15	5.87	9.87	12.60	1.34	2.48	3.26
2,4-Ditertiary butyl-phenol	0.00	0.00	0.00	0.98	0.89	0.87	0.55	0.84	0.06
Phenols	47.85	3.90	9.39	24.68	33.42	45.17	6.72	12.31	15.55

HTLWW samples were diluted into the same COD concentration before analysis

^a^“–” represents values below detection limit

### Quantitative identification of typical compounds of HTLWW samples

Table 3 summarizes the concentrations of typical organic fatty acids and total sugars in COD values in different HTLWW samples. Increased fatty acid production was observed with the increase of HTL temperature or residence time, which was also consistent with the decreased pH values as shown in Table 1. The dominant organic compound was acetic acid with the highest concentration 140 mg/g COD value for sample 320 °C–0.5 h. The content of sugars in sample 200 °C–0.5 h was 454 mg/g COD, while it sharply decreased to 175 mg/g COD for sample 230 °C–0.5 h and 22 mg/g COD for sample 320 °C–0.5 h.

**Table 3:** Summary of typical organics in COD values of different HTLWW samples

Name mg/g COD	170 °C–0.5 h	200 °C–0.5 h	230 °C–0.5 h	260 °C–0.5 h	290 °C–0.5 h	320 °C–0.5 h	200 °C–1 h	200 °C–2 h	200 °C–4 h
Lactic acid	9.25 ± 2.79	17.19 ± 3.11	89.48 ± 2.78	71.56 ± 8.99	88.87 ± 12.07	94.37 ± 3.25	41.72 ± 4.09	96.22 ± 14.78	91.00 ± 2.89
Acetic acid	5.46 ± 1.91	48.011 ± 9.02	80.04 ± 4.01	81.96 ± 1.78	106.34 ± 2.12	139.9 ± 4.01	47.59 ± 5.84	109.70 ± 2.13	96.95 ± 2.79
Propionic acid	2.47 ± 1.20	2.47 ± 0.01	5.39 ± 0.78	7.81 ± 1.98	13.37 ± 2.01	18.08 ± 0.02	3.36 ± 1.99	6.37 ± 2.05	6.53 ± 0.33
Isobutyric acid	–^a^	1.27 ± 0.65	1.17 ± 0.89	1.44 ± 1.78	1.86 ± 0.12	2.34 ± 1.03	–	1.82 ± 1.16	1.53 ± 1.89
*N*-Butyric acid	–	0.94 ± 0.02	1.53 ± 0.89	1.93 ± 0.79	3.51 ± 2.09	4.69 ± 2.56	1.12 ± 0.87	2.03 ± 1.13	1.84 ± 0.68
Isovaleric acid	2.73 ± 1.91	–	–	1.27 ± 1.78	1.38 ± 1.12	1.69 ± 0.91	–	–	1.68 ± 1.05
Pentanoic acid	–	–	1.17 ± 1.01	1.64 ± 0.65	–	–	1.21 ± 1.03	2.17 ± 0.78	1.84 ± 1.69
Sugars	383.26 ± 22.91	454.00 ± 56.08	174.65 ± 33.18	65.06 ± 3.82	30.57 ± 12.23	21.50 ± 0.17	323.40 ± 17.39	213.90 ± 22.90	107.10 ± 12.01
Total (in COD)	403.20 ± 30.72	524.00 ± 68.89	353.40 ± 43.54	232.7 ± 21.57	245.90 ± 31.76	282.50 ± 11.95	418.00 ± 14.26	432.01 ± 44.93	308.40 ± 33.49

^a^“–” represents values below detection limit and the detection limit was 0.01 mg/L for all the measured fatty acids

### Size distribution analysis of HTLWW samples

**Fig. 5:**
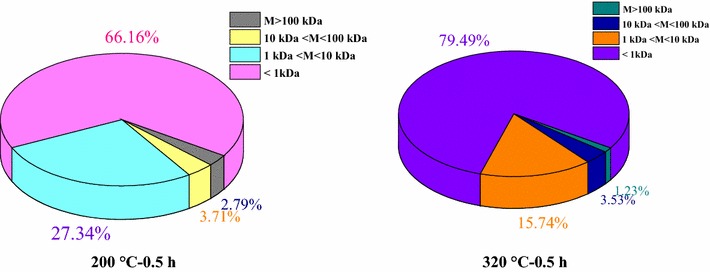
Percentages of molecular weight distributions of samples 200 °C–0.5 h 320 °C–0.5 h (in COD values)

**Fig. 6:**
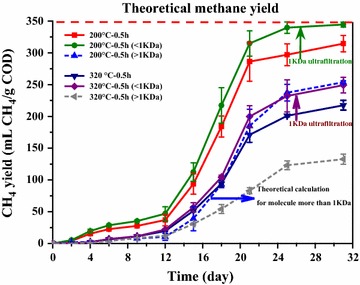
Methane yields of samples 200 °C–0.5 h and 320 °C–0.5 h before and after 1 KDa ultrafiltration

Organics with larger molecular weight need to be hydrolyzed before acidification and methanation, which is a relatively slow step [26] and therefore size distribution analysis of HTLWW might provide valuable information on the distribution of organics with different molecular sizes and their degradability. Size distribution of organics was for the first time used to characterize the HTLWW samples. Two samples (200 °C–0.5 h and 320 °C–0.5 h) were chosen since they had the highest difference in methane yields. Figure 5 and Table 4 show the COD values and their removal rates of samples 200 °C–0.5 h and 320 °C–0.5 h by membrane ultrafiltration with 100, 10, and 1 kDa membranes. It was obvious that organics with molecular size less than 1 kDa were dominant in the two HTLWW samples. Table 4 also shows that the organics with molecular size higher than 1 kDa were mainly distributed between 1 and 10 kDa. The methane yields of samples 200 °C–0.5 h and 320 °C–0.5 h after 1 kDa filtration were further evaluated in order to understand how the different molecular size of organics affected the methane yields of HTLWW, and the results are shown in Fig. 6. The methane yield of sample 200 °C–0.5 h ≤1 kDa was 345 mL CH_4_/g COD, which was close to the theoretical value (350 mL CH_4_/g COD), indicating this part of organics was easily biodegradable. Based on the methane yields of sample 200 °C–0.5 h and sample 200 °C–0.5 h ≤1 kDa, the methane yield of organics with molecular size higher than 1 kDa in sample 200 °C–0.5 h was calculated to be 253 mL CH_4_/g COD. For sample 320 °C–0.5 h, the methane yield of sample 320 °C–0.5 h ≤1 kDa was 239 mL CH_4_/g COD, and it was relative higher than that of sample 320 °C–0.5 h. However, the value was still much lower than the theoretical methane yield.

**Table 4:** COD values and removal rates before and after membrane ultrafiltration with 100, 10, and 1 kDa

Ultrafiltration	200 °C–0.5 h		320 °C–0.5 h	
	COD, g/L	R R, %	COD, g/L	R R, %
Raw WW	27.58 ± 2.89	–	24.33 ± 1.19	–
100 kDa	26.81 ± 1.91	2.79 ± 0.20	24.03 ± 0.97	1.23 ± 0.05
10 kDa	25.79 ± 0.71	6.49 ± 0.18	23.17 ± 1.11	4.77 ± 0.23
1 kDa	18.25 ± 1.03	33.82 ± 1.91	19.34 ± 0.45	20.51 ± 0.48

### Microbial community compositions

**Fig. 7:**
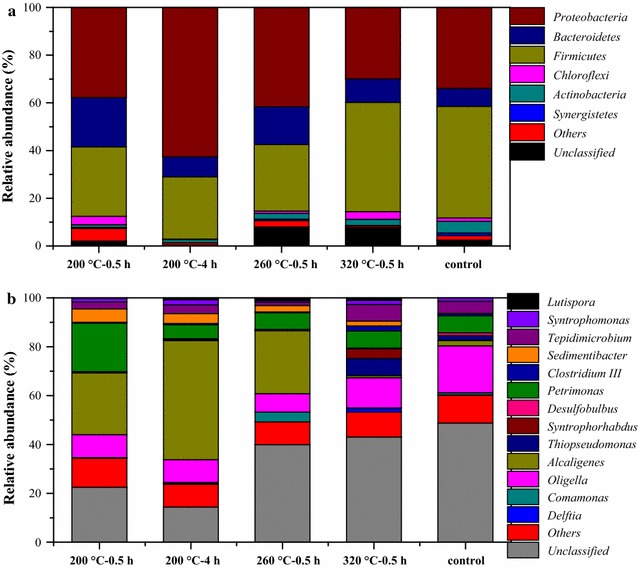
Classification of the sequences belonging to bacteria in different samples. **a** Phylum-level classification and **b** genus-level classification

As previously mentioned, the HTLWW contains various organics, and some of the organics were reported to be difficult to be biodegraded. The degradation of the organics to acetate and H_2_ by bacteria is crucial for methane production by methanogens. Therefore, it is necessary to understand the bacteria involved in the AD of HTLWW. As shown in Fig. 7a, all the samples were dominated by *Proteobacteria*, *Firmicutes*, and *Bacteroidetes*, which were commonly found in various biogas reactors [27–29]. The percentage of *Proteobacteria* in the sample 200 °C–4 h was 63%, which was obviously higher than that in the other samples (generally lower than 40%). It was further found that more than 70% of sequences in *Proteobacteria* were assigned to the genus *Alcaligenes* in sample 200 °C–4 h as shown in Fig. 7b. The abundance of genus *Petrimonas*, belonging to phylum *Bacteroidetes*, was 20% in sample 200 °C–0.5 h, which was much higher than that (<7%) in the other samples. The genus *Syntrophorhabdu*s (phylum *Proteobacteria*), capable of degrading phenols to acetate in obligate syntrophic associations with hydrogenotrophic methanogens [30], was enriched only in the sample 320 °C–0.5 h. Another genus *Sedimentibacter* (phylum *Firmicutes*), which could also convert phenols, was found to be present in all the samples (2–5.5%) except control sample [31].

**Fig. 8:**
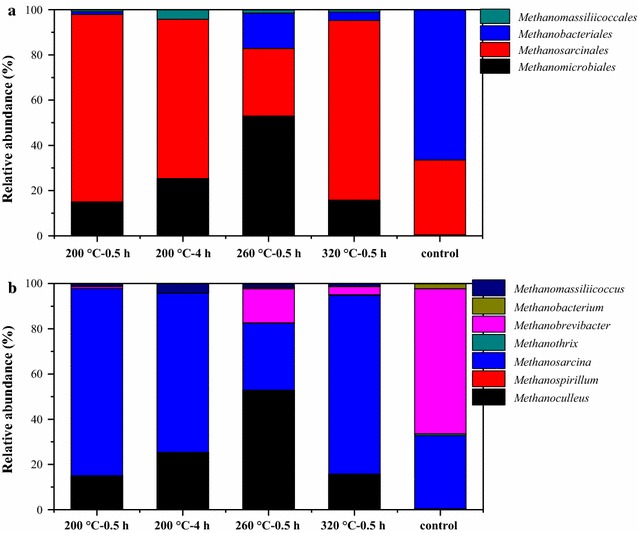
Classification of the sequences belonging to archaea in different samples. **a** Order-level classification and **b** genus-level classification

The archaeal community was also analyzed as shown in Fig. 8. The order *Methanosarcinales* was dominant in most of the samples. It is known that the order *Methanosarcinales* mainly contains genus *Methanosaeta* and *Methanosarcina* [32]; however, only genus *Methanosarcina* was found in all the samples. The hydrogenotrophic order *Methanomicrobiales* was also found to be dominant in all the samples except control, and the highest abundance (52%) was observed in sample 260 °C–0.5 h, indicating hydrogenotrophic methanogenesis as the predominant pathway.

### Energy recovery

**Fig. 9:**
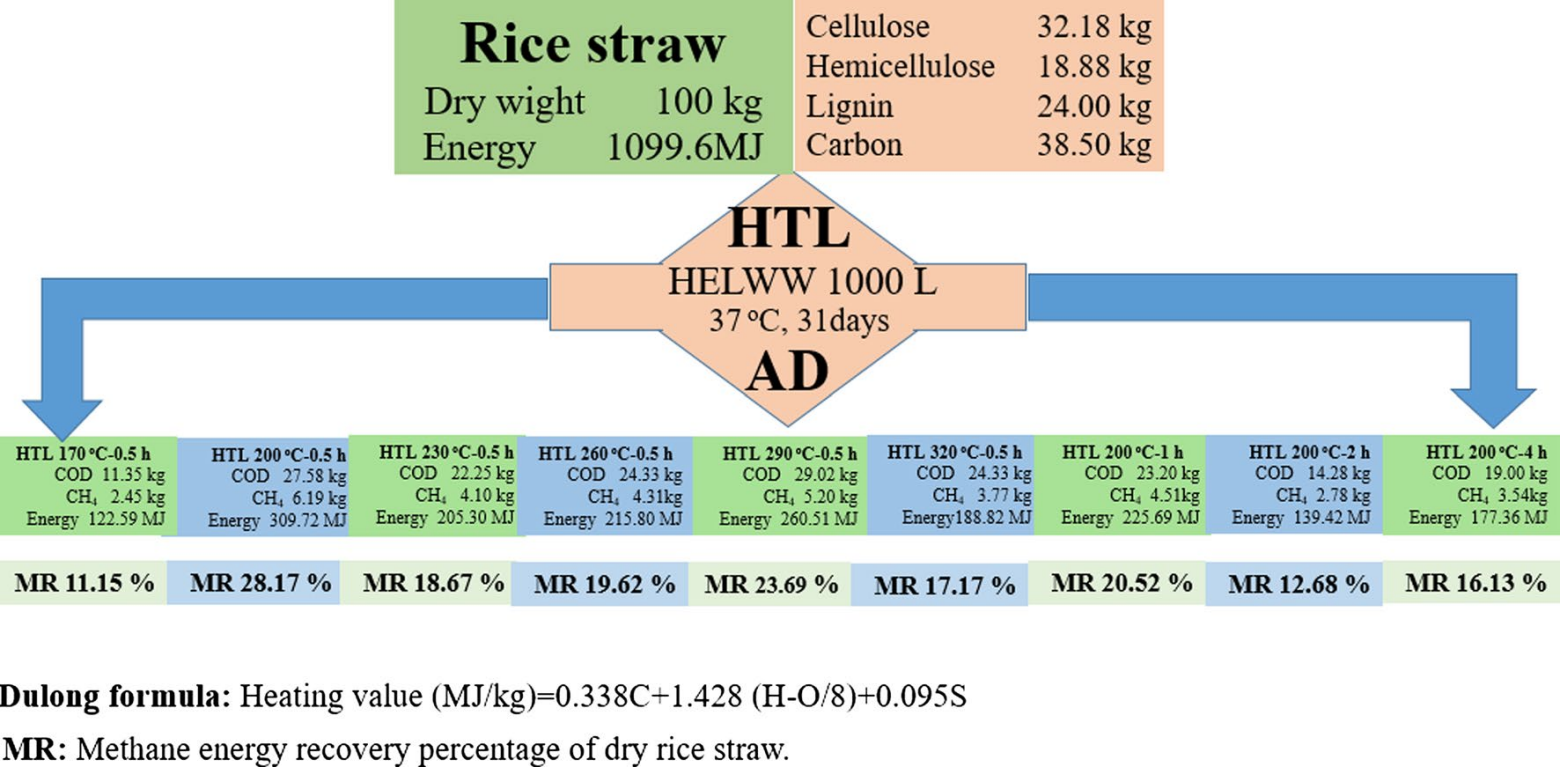
Energy recoveries of methane in the HTLWW acquired under different conditions

The energy recovery as methane from HTLWW obtained under different HTL conditions were calculated and shown Fig. 9. The recovered energy as methane from HTLWW through AD were in the range of 122.59 to 309.72 MJ per 100 kg of dry rice straw when HTL temperatures were increased from 170 to 320 °C and residence times were increased from 0.5 to 4 h at 200 °C, which equals to the energy recovery between 11.15 and 28.17% from rice straw.

## Discussion

The polytropic changes of COD and TOC values in Table 1 and Additional file 1: Figure S1 could be resulted from the hydrolyzation (decomposition) and repolymerization of different components in rice straw. A previous study showed that the TOC concentrations of HTLWW obtained from HTL of Pontianak tropical peat increased with the increase of temperature from 150 to 250 °C, but decreased in the range of 250–270 °C, possibly due to repolymerization [17]. Repolymerization reactions occurred along with this process and the products transferred into the solid phase [33]. The decrease of COD and TOC at 200 °C along with residence time might be related with repolymerization. The percentages of organics transferred into HTLWW were also calculated and are shown in Table 1, and it was in the range of 12–31%, which was lower than that (35–40%) for HTL of algae [34]. It could be mainly due to the different characteristics such as higher protein and carbohydrate contents in the algae utilized for HTL.

In a previous study, it was reported that around 44–61% of the COD in HTLWW obtained from HTL of algae was converted to methane [4]. In addition, the methane yield of 180 mLCH_4_/g COD was obtained from the HTLWW obtained by HTL of sewage sludge [35]. The methane yields obtained in the present study were between 217 mLCH_4_/g COD and 314 mLCH_4_/g COD, which was corresponded to 62–90% of the theoretical value (350 mL CH_4_/g COD). The conversion efficiencies of COD in the present study were higher than previously reported, which could be due to the different substrates for HTL; e.g., both algae and sewage sludge were rich in protein, while rice straw was mainly composed by carbohydrates. HTLWW obtained at 170 and 200 °C had relatively higher methane yields compared to the other conditions, indicating lower HTL temperature is crucial to achieve higher anaerobic biodegradability displays in Fig. 1. It was also demonstrated that the anaerobic biodegradability of HTLWW was lower when the temperature of hydrothermal processing increased although the biomass for HTL was quite different [36]. Insufficient conversion indicating recalcitrant organics was present in all HTLWW samples, especially the samples obtained at high HTL temperature (e.g., 320 °C). Therefore, characterization of the different HTLWW samples was conducted to understand how the HTL conditions affected the biodegradability of HTLWW.

All HTLWW samples had absorption peak in the range of 320–250 nm. It was reported that many degradation products of sugars (from hemicellulose and cellulose) and lignin had absorption peak in the range of 320–250 nm [37]. For the hydrolysates of sugars, the peaks at 278 and 284 nm corresponded to furfural and the mixture of furan and 5-hydroxymethylfurfural (HMF), respectively [37]. A rapid method for determination of furfural using UV spectroscopy also showed furfural compound had a peak absorbance at 276 nm [38]. Besides, UV_254_ provides an indication of the concentration of unsaturated bonds (double and triple) structures and aromatic ring matters, which was difficult to be biodegraded [39]. The UV_254_ to COD ratio values are listed in Table 1. It could be confirmed that the absorption at the range of 320–250 nm was mainly attributed to the sugar degradation products of unsaturated bonds instead of lignin. This is because sample 320 °C–0.5 h had higher phenols but the UV_254_/COD ratio of sample 320 °C–0.5 h was the lowest (lignin degradation products would be discussed in following GC–MS analysis section). It was also reported that sugars could be converted into furan derivatives, HMF, and furfural products at around 200 °C [17, 40]. Particularly, as Fig. 3b shows, sample 200 °C–1 h had a high absorption peak at 278 nm, and it also had relatively lower methane yield in Fig. 2. Nevertheless, the reason for lower methane yields of samples 320 °C–0.5 h and 200 °C–1 h was still not clear and other methods were needed to identify other organics to better understand the AD of HTLWW.

It further confirmed the conclusion that sugar degradation products attributed to the absorption at the range of 320–250 nm when comparing GC–MS and UV results. The highest amounts of phenols (phenol, ethyl-phenol, and methoxy-phenol) were detected in sample 320 °C–0.5 h. In particularly, the highest content of furans was confirmed in the sample 200 °C–1 h, which was exactly consistent with the UV analysis. Besides, the content of furans in hydrolysates was used as a predictor for the toxicity of the hydrolysates from the HTL of hemicellulose [37], and this could be one of the reasons that the methane yield of sample 200 °C–1 h was much lower than that of sample 200 °C–0.5 h. For samples 320 °C–0.5 h and 200 °C–4 h, relative higher concentrations of furans, ketones, and phenols might contribute to their lower methane yields compared to other samples [41]. It still needs other characterizations to directly explain samples such as 200 °C–0.5 h with higher methane yield.

As Table 3 shows, 200 °C–0.5 h had the highest total sugars and short-chain organic acids content (524 mg/g COD) especially the sugars content (454 mg/g COD). It could be one reason for the highest methane yield obtained from sample 200 °C–0.5 h since sugars and short-chain organic fatty acids were relatively easier to be biodegraded by AD [42, 43]. It should be noted that sample 200 °C–2 h had higher amount of sugars and organic fatty acids (432 mg/g COD) compared to sample 170 °C–0.5 h (403 mg/g COD); however, it had significantly lower methane yield (259 mL CH_4_/g COD) compared to sample 170 °C–0.5 h (302 mL CH_4_/g COD), which indicated that other easily degradable organics might be present in 170 °C–0.5 h but not quantified in the present study. In addition, the relatively higher content of refractory compounds (furans, similar value to 200 °C–1 h Table 2) was also found in sample 200 °C–2 h as mentioned before, which could be one reason for the relatively lower methane yield.

A new vision from size fractionation of organics in HTLWW on AD was tested. Comparing with sample 200 °C–0.5 h, sample 320 °C–0.5 h contained more organics with molecular size less than 1 kDa (66.2% vs 79.5%). This indicated that there were more high molecular size compounds presented in sample 200 °C–0.5 h and higher temperature facilitated the decomposition of high molecular size compounds [44]. As Fig. 5 displays, lower methane yield of sample 200 °C–0.5 h compared to theoretical value was mainly attributed to the presence of refractory organics with molecular size higher than 1 kDa. It was obvious for sample 320 °C–0.5 h that the refractory organics were still present in the organics with molecular size lower than 1 kDa, which was different from the sample 200 °C–0.5 h. The result was consistent with GC–MS analysis since more phenols and ketones which were difficult for biodegradation were detected in sample 320 °C–0.5 h compared to sample 200 °C–0.5 h. The molecule weights of eighteen ketones and six phenols identified in Table 2 were around 200 Da whose molecular size was lower than 1 kDa and distributed in the phase with molecular size lower than 1 kDa. The methane yield of organics with molecular size higher than 1 kDa in sample 320 °C–0.5 h was also calculated and it was only 132 mL CH_4_/g COD, indicating the presence of refractory organics with molecular size higher than 1 kDa in sample 320 °C–0.5 h.

Although there were several studies focusing on the methane production from HTLWW originated from different biomasses [4, 35], the microbial community was seldom investigated. Only our previous study made such analysis and found *Firmicutes* was dominant in the microbial community during AD of HTLWW [10], which was consistent with the present study. It should be noted that *Proteobacteria* was also dominant in the samples of the present study. E.g, the relative abundance of *Proteobacteria* was 63% in the sample 200 °C–4 h and more than 70% of the *Proteobacteria* was assigned to the genus *Alcaligenes* as shown in Fig. 7b. Previous literature showed that the genus *Alcaligenes* is strictly aerobic and some strains are capable of anaerobic respiration in the presence of nitrate or nitrite [45, 46]. The relative abundance of *Alcaligenes* in the control sample was very low (2.2%), while it was dominant (between 25 and 50%) in samples 200 °C–0.5 h, 200 °C–4 h, and 260 °C–0.5 h. In addition, a previous study also detected the genus *Alcaligenes* in the AD of mixed-microalgal biomass [47]. Therefore, it seems that the genus *Alcaligenes* played an important role in the AD. However, the functional roles of genus *Alcaligenes* in AD needs further elucidation. It should be noted that the abundance of genus *Alcaligenes* in sample 320 °C–0.5 h was extremely low (around 1%). Tables 2 and 3 show that the concentrations of furfurals and sugars in samples 200 °C–0.5 h, 260 °C–0.5 h, and 200 °C–4 h were much higher than that in sample 320 °C–0.5 h, which might be related with the enrichment of the genus *Alcaligenes*. Besides, it has been reported the microorganisms in genus *Petrimonas* (belonging to phylum *Bacteroidetes*) could ferment sugars to produce acetate [48], and its abundance in sample 200 °C–0.5 h could be related with the higher sugar content (454 mg/g COD) compared with the other samples. The genus *Syntrophorhabdu*s (phylum *Proteobacteria*) could be related with the much higher content of phenols in HTLWW at 320 °C–0.5 h compared to the other samples as shown in Table 2.

In addition, the abundance of genus *Sedimentibacter* decreased with the increase of HTL temperature. For example, the abundance of *Sedimentibacter* was 5.5% in sample 200 °C–0.5 h, while it decreased to 1.9% in sample 320 °C–0.5 h. The above results showed that some known bacteria relating with sugar and phenol degradations were enriched during the AD of HTLWW. Considering the complex organics present in HTLWW, more functional versatile bacteria should be enriched. There might be two reasons. On the one hand, the functional properties of the isolated bacteria might not be fully explored, such as the genus *Alcaligenes* as previously mentioned. On the other hand, there were relatively high percentages (between 14 and 50%) of unclassified sequences in genus level in the samples, which were unknown bacteria and remain to be investigated.

*Methanosarcina* was found in all the samples, while *Methanosaeta* was not for archaeal community as shown in Fig. 8. It could be due to the fact that *Methanosaeta* are strictly aceticlastic methanogens and are sensitive to the environmental conditions, while *Methanosarcina* can mediate both aceticlastic and hydrogenotrophic methanogenesis [49]. In all the HTLWW samples, various kinds of organics were present, some of which may be toxic to methanogens, and therefore only the metabolically versatile *Methanosarcina* could survive. Previous study also showed that hydrogenotrophic methanogens are more tolerant to the changes of environmental conditions compared to aceticlastic methanogens [50]. As mentioned before, hydrogenotrophic methanogens are important for the degradation of certain organics (phenols) by syntrophic associations with bacteria [30]. The above results clearly showed that *Methanosarcina* instead of *Methanosaeta* was dominant for the AD of HTLWW at various conditions.

The energy recoveries as methane from rice straw were between 11.15 and 28.17% (Fig. 9), and they were lower than that reported in a previous study where the energy recovery as high as 50% was obtained [36]. It could be due to the fact that relatively lower percentages of organic components were transferred to aqueous phase for HTL of rice straw in the present study (between 12.11 and 29.42% in COD value as shown in Table 1). However, in the above-mentioned study, food waste was used and the organics transferred into aqueous phase by HTL were in the range of 35 to 87%.

The combination of HTL and AD can achieve efficient utilization of biomass for biofuels production in the form of bio-oil and biogas. The present study clearly showed that the HTL conditions significantly affected the compositions of HTLWW, and thereby resulted in variation of microbial community compositions in AD and finally affected the methane potentials of HTLWW. The increase of HTL temperature (higher than 230 °C) and residence time (longer than 1 h) were not beneficial for biogas production from HTLWW, since hard degradable or even inhibitors like phenols, furan, and 5-hydroxymethylfurfural were produced. Therefore, very high HTL temperature and long residence time should be avoided for HTL of rice straw and similar lignocellulose biomass if HTLWW will be treated by AD for methane production. In addition, separation of the inhibitory compounds before AD might also be applied in order to increase the biogas production from HTLWW. For example, several methods are developed in order to separate furans, phenols, and ketones in our group [51, 52], which could not only get high value chemicals but also might improve the anaerobic degradability of HTLWW. Although rice straw was used in the present study, the results obtained might also be transferable to other HTLWW samples obtained from HTL of lignocellulose materials.

## Conclusions

The present study showed that HTL temperature and residence time obviously affected the anaerobic biodegradability of HTLWW. The highest and lowest methane yields were 314 and 217 mL CH_4_/g COD obtained from sample 200 °C–0.5 h and sample 320 °C–0.5 h, respectively. The methane production potential was related to different contents of hard biodegradable organics (furans and phenols) and easily biodegradable organics (sugars and volatile fatty acids) in the samples. The study also showed that the organics with molecular size less than 1 kDa for sample 200 °C–0.5 h could be almost fully converted to methane (methane yield 345 mLCH_4_/g COD). However, organics with molecular size higher than 1 kDa for 200 °C–0.5 h contained recalcitrant organics and could not be fully converted as the methane yield showed 253 mLCH_4_/g COD. In addition, for sample 320 °C–0.5 h, the organics with molecular size both less (methane yield 249 mL CH_4_/g COD) and higher (methane yield 132 mLCH_4_/g COD) than 1 kDa had lower methane yields compared to those for sample 200 °C–0.5 h.

Further microbial community analysis showed that different microbial community compositions were established during the AD with different HTLWW samples, which was correlated with the different organic compositions. The higher *Petrimonas* abundance was consistent with the higher content of sugars in sample 200 °C–0.5 h and the enrichment of genus *Syntrophorhabdu*s was related with the highest content of phenols in sample 320 °C–0.5 h. Besides, *Methanosarcina* instead of *Methanosaeta* was dominant for the AD of complicated HTLWW at various conditions.

## Methods

### Preparation of HTLWW

Rice straw with particle size ranging between 0.2 and 1.0 mm was for HTL. The characteristics of rice straw are shown in Additional file 1: Table S1. HTL of rice straw was performed in a 250-mL completely mixed stainless steel (316L) reactor (Yan Zheng experiment instrument Co., Ltd, Shanghai, China). The temperature of the reactor was controlled with a programmable temperature controller and a digital thermometer. In a typical run, 15 g of rice straw and 150 mL MilliQ water were loaded into the reactor to obtain the water/biomass ratio of 10:1 [53]. Then the reactor was sealed and heated to the desired temperature (170, 200, 230, 260, 290, and 320 °C) with the residence time 0.5 h. In addition, different residence times (0.5, 1, 2, and 4 h) were also conducted for the HTL at 200 °C. After reaching the desired residence time, the reactor was removed from the heater and quenched rapidly in a water bath to stop the reactions. The solid and liquid products were collected after depressurization, and liquid products (HTLWW) were separated from solid products by a vacuum buchner funnel through 0.45-μm membranes. HTLWW samples were stored in refrigerator at −20 °C for further utilization.

### Methane potentials of HTLWW

Batch experiments were conducted to determine the methane potentials of HTLWW obtained from different HTL conditions. The experiments were conducted in 118-mL serum bottles with 60 mL working volume. 15 mL inoculum and 45 mL BA medium containing HTLWW and 5 g/L NaHCO_3_ were added to each bottle, and the initial COD value of all assays were controlled at 0.75 g/L by adding different amounts of HTLWW to the BA medium. pH values were adjusted to 7.5 by the addition of 2 M NaOH and 2 M HCl. All the bottles were flushed with N_2_ to remove oxygen before incubation, and then sealed with butyl rubber stoppers and aluminum screw caps. The bottles were placed in an incubator with temperature controlled at 37 °C. The inoculum used in the study was obtained from a lab-scale biogas reactor treating sewage sludge with TS 17.4 g/L, VS 12.9 g/L, and pH 7.2. All the experiments were conducted in triplicates. The bottles with only inoculum were used as control.

### Size fractionation of HTLWW

Two HTLWW samples 200 °C–0.5 h and 320 °C–0.5 h were chosen based on their methane potentials (the maximum and minimum values) to determine the effects of size fractionation of organics in HTLWW on methane potentials. Size fractionation of organics in HTLWW was carried out in a dead-end batch ultrafiltration apparatus. The apparatus was consisted of 400-mL stirred ultrafiltration cell (model 8400, Amicon, Belford, MA), a nitrogen gas tank (pressure: 200 kPa), and membrane disks (Millipore, Billerica, MA) with diameter of 76 mm. The MW cutoffs of membrane disks used in the study were 1, 10, and 100 kDa, (PLAC, PLGC, and PLHK Millipore, Billerica, MA). HTLWW samples were filtered and collected, and then they were stored at 4 °C for further analysis and methane potential tests. Based on the results from size fractionation, the methane potentials of HTLWW after 1 kDa filtration were determined to understand how the size fractionation of organics affected the methane potential of different HTLWW samples.

### Microbial community analysis

Four samples (200 °C–0.5 h, 260 °C–0.5 h, 320 °C–0.5 h and 200 °C–4 h) were collected when the cumulative methane production achieved maximum values in the batch experiments for methane potentials test. Sample 200 °C–0.5 h had the highest methane yield, while sample 320 °C–0.5 h had the lowest yield. Temperature 260 °C was the medium temperature of 200 and 320 °C and the methane yield value was medial. Besides, since the methane yields were not significantly changed at 200 °C when the HTL residence time was increased from 1 to 4 h, the sample 200 °C–4 h was chosen. Total genomic DNA was extracted from each sample using QIAamp DNA Stool Mini Kit (QIAGEN, 51504). The quantity and purity of the extracted DNA were checked by Nanodrop 2000 (Thermo Scientific, USA). PCR was then conducted with the universal primers 515 F (5′-GTGCCAGCMGCCGCGGTAA-3′) and 806 R (5-GGACTACHVGGGTWTCTAAT-3′) targeting both bacteria and archaea according to previous studies [49, 54]. The PCR products were purified, quantified, and used for barcoded libraries preparation and then sequenced on an Illumina Miseq platform according to the standard protocols. The obtained sequences were deposited in the NCBI sequence read archive database (Accession ID: SUB2302564). The low-quality sequences without exact matches to the forward and reverse primers, with length shorter than 100 bp, and containing any ambiguous base calls, were removed from the raw sequencing data by RDP tools (http://pyro.cme.msu.edu/). Chimeras were removed from the data using the Find Chimeras web tool (http://decipher.cee.wisc.edu/FindChimeras.html). The numbers of sequences after quality filtration from different samples are shown in Additional file 1: Table S2. The high-quality sequences were phylogenetically assigned to taxonomic classifications by RDP Classifier with a confidence threshold of 80%.

### Analytical methods

COD was measured according to APHA (APHA, 1995). Total organic carbon (TOC) was analyzed by a TOC analyzer (TOC-L CPH, Shimadzu, Japan). pH value was measured using a pH meter (FE20, Mettler Toledo, Switzerland). UV spectra and UV_254_ were measured by TU-1901 ultraviolet–visible light spectrophotometer which was made by general analysis instrument co., LTD of Beijing.

3-Dimensional excitation–emission matrix (EEM) fluorescence spectroscopy was measured for excitation wavelengths of *λ*ex = 240–600 nm at 3-nm increments across an emission range of *λ*em = 280–550 nm at 3-nm intervals by Fluorescence Spectrophotometer (Horiba, Japan). Inner filter effect was minimized by diluting the samples until the absorbance at wavelength of 254 nm was smaller than 0.05/cm. Data were processed using the FL Toolbox v1.91 for MATLAB 7.0 and presented as an EEM. Before analysis, the EEM correction process was carried out consisting of blank EEM subtraction, scatter line removal, application of excitation and emission correction factors, correction for inner filter effects, and normalization to Raman Units.

GC–MS (Focus DSQ, Thermoelectron, America) was used to characterize the chemical compositions of HTLWW samples. Gas chromatography was performed on a 30-m HP-INNOWax quartz capillary column with 0.25 mm inner diameter (I.D.) and 0.25 μm film thickness with injection temperature of 250 °C. The column was initially held at 60 °C for 2 min and heated to 250 °C and held for 10 min. Helium was served as the carrier gas (1.0 mL/min). A NIST Mass Spectral Database (https://www.nist.gov/srd/nist-standard-reference-database-1a-v14) was used for compound identification.

The concentrations of volatile fatty acids were determined by GC (Shimadzu G2010) with a flame ionization detector, and the lactic acid concentrations were measured by high-performance liquid chromatography. Detailed information about the above analysis can be found in our previous studies [10].

### Calculation of methane production efficiency

COD degradation efficiencies were calculated based on measured methane volumes in the fermentation serum bottles as well as on the COD input (0.045 g COD) equivalent of methane volume. The percentage of methanogenesis was calculated using Eq. (1), where V_STP-CH4_ is the cumulative methane production volume from external carbon sources. It is calculated by subtracting three average cumulative productions observed in control bottles from the average cumulative production observed in the bottles fed with HTLWW as carbon source, and V_0-CH4_ is the theory methane volume provided by mass of organic matter in each trial. The maximum theoretical methanogenic potential was calculated as 350 mL of CH_4_ generated per gram of removed COD [55]. All values of methane yield reported at standard temperature and pressure (STP) throughout the study.

1 $${\text{CH}}_{ 4} (\% ) = \frac{{V_{{{\text{STP}} - {\text{CH}}_{ 4} }} }}{{V_{{0 - {\text{CH}}_{ 4} }} }} \times 100\%.$$

## Additional file

**Additional file 1: Table S1.** Characteristics of rice straw and the following line is standard error of each value; **Table S2.** Number of the high-quality sequences; **Figure S1.** COD, TOC and pH values of HTLWW samples under different HTL conditions; **Figure S2.** Comparison of methane production potentials of samples 200 °C–0.5 h, 260 °C–0.5 h and 200 °C–4 h.

## Abbreviations

AD: anaerobic digestion
EEM: excitation–emission matrix
HMF: hydroxymethylfurfural
HTL: hydrothermal liquefaction
HTLWW: hydrothermal liquefaction wastewater
STP: standard temperature and pressure
TOC: total organic carbon
