# Supplementary material for: Methane potentials of wastewater generated from hydrothermal liquefaction of rice straw: focusing on the wastewater characteristics and microbial community compositions
Source: Biotechnol Biofuels. 2017 May 31;10:140. doi: 10.1186/s13068-017-0830-0 (PMC5452606; doi:10.1186/s13068-017-0830-0)
Supplement: Supplementary file 1 — Additional file 1: Table S1. Characteristics of rice straw and the following line is standard error of each value; Table S2. Number of the high-quality sequences; Figure S1. COD, TOC and pH values of HTLWW samples under different HTL conditions; Figure S2. Comparison of methane production potentials of samples 200 °C–0.5 h, 260 °C–0.5 h and 200 °C–4 h. [file 13068_2017_830_MOESM1_ESM.docx]

Supporting information

**Methane potentials of wastewater generated from hydrothermal liquefaction of rice straw: Focusing on the wastewater characteristics and microbial community compositions**

Huihui Chen, Cheng Zhang, Yue Rao, Yuhang Jing, Gang Luo*, Shicheng Zhang*

Shanghai Key Laboratory of Atmospheric Particle Pollution and Prevention (LAP^3^), Department of Environmental Science and Engineering, Fudan University, Shanghai 200433, China

^*^ Corresponding author:

Gang Luo: [gangl@fudan.edu.cn](mailto:gangl@fudan.edu.cn), +86 65642297

Shicheng Zhang: [zhangsc@fudan.edu.cn](mailto:zhangsc@fudan.edu.cn), +86 65642297

Figure S1 COD, TOC and pH values of HTLWW samples under different HTL conditions.

Figure S2 Comparison of methane production potentials of samples 200 ^o^C-0.5 h, 260 ^o^C -0.5 h and 200 ^o^C -4 h.

Table S1 Characteristics of rice straw and the following line is standard error of each value.

| **Chemical composition (wt % ^a^)** | | | **Moisture** | **Ash** | **HHV** | **Elemental composition** | | | |
| --- | --- | --- | --- | --- | --- | --- | --- | --- | --- |
|  |  |  | **(wt %)** | **(wt %)** | **(wt %)** | **(wt %)** | | | |
| Cellulose | Hemicellulose | Lignin |  |  |  | C | H | O | N |
| **32.18**±1.78 | **18.88**±0.96 | **24.00**±1.59 | **17.82**±2.02 | **8.2**±0.78 | **11.07**±0.09 | **38.5**±0.99 | **5.6**±0.16 | **55.3**±1.24 | **0.6**±0.02 |

^a^: wt % of dry basis.

**The percentage organics (in COD values) in HTLWW measuring method:**

$$\text{Molecular formula}=\text{C}_{\frac{0.385}{12}}\text{ H}_{0.56}\text{ O}_{\frac{0.56}{16}}\text{ N}_{\frac{0.006}{14}}\text{ }$$

$\begin{matrix} \text{COD}_{\text{Input}}=15\times(\frac{0.385\times2\times16}{12}+\frac{0.0554\times16}{2}-0.553+\frac{0.006}{14}\times2\times16=14.06 \text{g} \\ \end{matrix}$ S(1)**$\text{COD \%}_{\text{Percentage in HTLWW}}=\frac{\text{COD}_{\text{Value in HTLWW}}\times0.15\text{L}}{14.06 \text{g}}\times100\%$** S(2)

**The equation for calculating theoretical methane yield**

$$CH_{4}+2O_{2}=CO_{2}+2H_{2}O$$

$V_{CH_{4}/\text{gCOD}}=\frac{1\times22.4L}{16\times4}\times1000=350 \text{mL }CH_{4}/gCOD$ S(3)

| Sample ID | Number of high quality sequences | Average length (bp) |
| --- | --- | --- |
| 200 ^o^C -0.5 h | 22775 | 275 |
| 200 ^o^C -4 h | 26020 | 275 |
| 260 ^o^C -0.5 h | 21627 | 275 |
| 320 ^o^C -0.5 h | 22257 | 275 |
| Control | 35159 | 275 |

Table S2 Number of the high quality sequences
